# Antitoxin. I. The Production of Diphtheria Toxin

**Published:** 1895-04

**Authors:** William Hallock Park


### ANTITOXIN.[1]

### I. The Production of Diphtheria Toxin.

BY WILLIAM HALLOCK PARK, M.D.

Before describing the process of preparing the diphtheria antitoxin it will be well to state briefly our present views upon the relation of the diphtheria bacilli to diphtheria. Recent investigation has in no way weakened our belief that true diphtheria, and by that I mean a pseudo-membranous inflammation which gives us clinically the characteristic picture which we have always recognized as diphtheria, is primarily caused by the bacillus diphtheria of Lœffler. We know now, indeed, that the virulent bacillus may be taken into the mouth, and may even multiply and remain in the throat secretions for days and weeks without giving rise to diphtheria; but under conditions favorable to the development of diphtheria these bacilli are perfectly able to start up disease either in the person's throat where they have been lying dormant or in the throats of others to which they have been transferred.

The preparation of the toxin of the diphtheria bacillus for the purpose of injecting animals in order that they may produce in their bodies the diphtheria antidote—the antitoxin—has been so fully and so often described that I will but briefly touch on certain practical points. The diphtheria bacilli should be obtained from a number of severe cases of diphtheria, and should be tested as to their virulence by injecting them in guinea-pigs. From the number tested four or five of the most virulent cultures, which have been derived from different cases, should be selected to inoculate the bouillon for making the toxin. Experience has taught that bacilli from different sources having equal virulence in guinea-pigs do not always produce the same amount of toxin in bouillon. It is thus better not to inoculate all the flasks from one culture. It has also been found that different cultures vary greatly in the length of time the bacilli retain their full virulence. In making the original stock cultures it is well to inoculate the bouillon in each of the

[1] Papers read before the Veterinary Medical Association of New York County, March 5, 1895.

tubes from a number of colonies from the agar plates, the cultures thus obtained being more apt to be virulent. The flasks used for the preparation of toxin may be either of the ordinary shapes or may be flat-bottomed, with tubes for the inlet and exit of air. I have found that the bacilli grown in a thin layer of bouillon in ordinary flasks loosely stoppered with cotton have produced toxins about as strong and nearly as quickly as those grown in the bouillon to which a constant supply of moistened air has been added. Toxins, of which one-tenth of a cubic centimetre was sufficient to kill a 250-gramme guinea-pig were produced in from two to four weeks. In some cases the toxins were twice the above strength. If to an old culture ½ per cent. carbolic acid is added and the bacilli, after being killed, are allowed to remain soaking in the bouillon, the amount of toxin will be found to gradually increase. The dead bacilli give up some of the toxin contained in their bodies to the bouillon. In order to take advantage of this fact the bacilli are left with the toxin after ½ per cent. carbolic acid has been added until the toxins are desired for use. At times when the strong toxins were scanty the dead bacilli were not filtered out, but injected along with the weaker toxin. The only evil effect noticed was the liability to produce slight local abscesses in the horses.

---

